# 2-(1-Adamant­yl)-1,3-diphenyl­propan-2-ol

**DOI:** 10.1107/S1600536810030047

**Published:** 2010-07-31

**Authors:** Eva Babjaková, Marek Nečas, Robert Vícha

**Affiliations:** a Department of Chemistry, Faculty of Technology, Tomas Bata University in Zlin, Nám. T. G. Masaryka 275, Zlín,762 72, Czech Republic; b Department of Chemistry, Faculty of Science, Masaryk University, Kamenice 5, Brno-Bohunice, 625 00, Czech Republic

## Abstract

In the title compound, C_25_H_30_O, the adamantane cage consists of three fused cyclo­hexane rings in classical chair conformations, with C—C—C angles in the range 107.15 (9)–111.55 (9)°. The dihedral angle between the benzene rings is 46.91 (4)° and the conformation is stabilized by a weak intra­molecular C—H⋯π inter­action.

## Related literature

For the preparation and spectroscopic properties of the title compound, see: Vícha *et al.* (2006 ▶). For related structures, see: Vaissermann & Lomas (1997 ▶); Vícha & Nečas (2010 ▶).
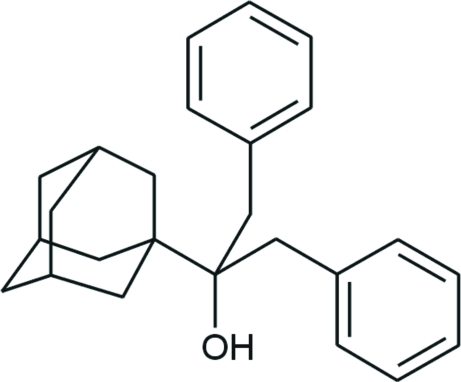


## Experimental

### 

#### Crystal data

C_25_H_30_O
*M* *_r_* = 346.49
Monoclinic, 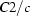

*a* = 24.2808 (10) Å
*b* = 6.3978 (2) Å
*c* = 25.2555 (14) Å
β = 106.183 (5)°
*V* = 3767.8 (3) Å^3^
*Z* = 8
Mo *K*α radiation
μ = 0.07 mm^−1^
*T* = 120 K
0.40 × 0.30 × 0.20 mm

#### Data collection

Oxford Diffraction Xcalibur diffractometer with a Sapphire2 (large Be window) detector
Absorption correction: multi-scan (*CrysAlis RED*; Oxford Diffraction, 2009 ▶) *T*_min_ = 0.965, *T*_max_ = 1.000
17425 measured reflections
3315 independent reflections
2381 reflections with *I* > 2σ(*I*)
*R*_int_ = 0.024

#### Refinement

*R*[*F* ^2^ > 2σ(*F* ^2^)] = 0.033
*wR*(*F* ^2^) = 0.080
*S* = 0.96
3315 reflections
236 parameters
H-atom parameters constrained
Δρ_max_ = 0.20 e Å^−3^
Δρ_min_ = −0.21 e Å^−3^

### 

Data collection: *CrysAlis CCD* (Oxford Diffraction, 2009 ▶); cell refinement: *CrysAlis RED* (Oxford Diffraction, 2009 ▶); data reduction: *CrysAlis RED*; program(s) used to solve structure: *SHELXS97* (Sheldrick, 2008 ▶); program(s) used to refine structure: *SHELXL97* (Sheldrick, 2008 ▶); molecular graphics: *ORTEP-3* (Farrugia, 1997 ▶) and *Mercury* (Macrae *et al.*, 2008 ▶); software used to prepare material for publication: *SHELXL97*.

## Supplementary Material

Crystal structure: contains datablocks global, I. DOI: 10.1107/S1600536810030047/zl2292sup1.cif

Structure factors: contains datablocks I. DOI: 10.1107/S1600536810030047/zl2292Isup2.hkl

Additional supplementary materials:  crystallographic information; 3D view; checkCIF report

## Figures and Tables

**Table 1:** Hydrogen-bond geometry (Å, °) *Cg*1 is the centroid of C20–C25 ring.

*D*—H⋯*A*	*D*—H	H⋯*A*	*D*⋯*A*	*D*—H⋯*A*
C14—H14⋯*Cg*1	0.95	2.70	3.3172 (13)	123

**Fig. 1.**
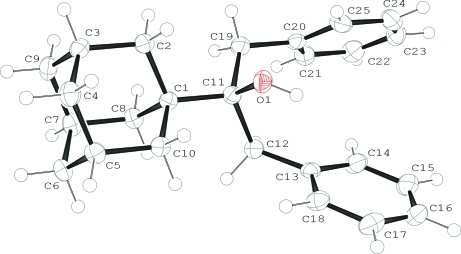
Ellipsoid plot (50% probability) of the asymmetric unit. Hydrogen atoms are represented as spheres of arbitrary size.

**Fig. 2.**
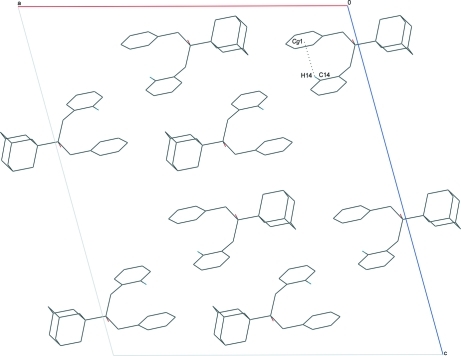
Crystal packing viewed along the b-axis. Intramolecular C—H···π interactions are shown as dotted lines. Cg1 is the center of gravity of C20–C25. H-atoms (except those which are involved in C—H···π interactions) have been omitted for clarity.

## supplementary crystallographic information

### Comment

In the title compound, both benzene rings (C13–C18 and C20–C25) are essentially planar with maximum deviations from their respective best planes of 0.0053 (12) Å for C17 and 0.0073 (12) Å for C25. The angle between the best planes of these rings is 46.97 (3)°. The torsion angles that describe the arrangement of the two benzyl substituents and the adamantane cage – C2—C1—C11—O1, C1—C11—C12—C13, C1—C11—C19—C20, C11—C12—C13—C14 and C11—C19—C20—C21 – are 63.42 (11)°, -134.29 (10)°, -174.22 (10)°, -104.43 (13)°, and 107.99 (13)°, respectively. The conformation of the molecules in the crystal is stabilized by a weak C—H···π interaction, C14—H14···*Cg*1 (*Cg*1 is the centre of gravity of C20–C25), with a C14···*Cg*1 distance of 3.3172 (13) Å (see Fig. 2 and Table 1). In analogy to the previously published structure of 1-adamantyl(diphenyl)methanol (Vícha & Nečas, 2010), no H-bonds were observed in the crystal packing. The shortest distance between two adjacent O-atoms is 4.7666 (11) Å. Surprisingly, the more strained molecules of di(1-adamantyl)(2,5-diisopropylphenyl)methanol with two bulky adamantane cages form O—H···O linked dimers in the solid state (Vaissermann & Lomas, 1997).

### Experimental

The title compound was isolated from a complex mixture obtained from the reaction of adamantane-1-carbonyl chloride with benzylmagnesium chloride as described previously (Vícha *et al.*, 2006). The crystal used for data collection was grown by slow evaporation of a solution in hexane at room temperature.

### Refinement

Hydrogen atoms were positioned geometrically and refined as riding using standard *SHELXTL* constraints, with their *U*_iso_ values set to 1.2U_eq_ of that of their parent atoms.

### Crystal data

**Table:**

C_25_H_30_O	*F*(000) = 1504
*M**_r_* = 346.49	*D*_x_ = 1.222 Mg m^−^^3^
Monoclinic, *C*2/*c*	Melting point: 396 K
Hall symbol: -C 2yc	Mo *K*α radiation, λ = 0.71073 Å
*a* = 24.2808 (10) Å	Cell parameters from 6762 reflections
*b* = 6.3978 (2) Å	θ = 3.0–27.2°
*c* = 25.2555 (14) Å	µ = 0.07 mm^−^^1^
β = 106.183 (5)°	*T* = 120 K
*V* = 3767.8 (3) Å^3^	Block, colourless
*Z* = 8	0.40 × 0.30 × 0.20 mm

### Data collection

**Table:**

Oxford Diffraction Xcalibur diffractometer with a Sapphire2 (large Be window) detector	3315 independent reflections
Radiation source: Enhance (Mo) X-ray Source	2381 reflections with *I* > 2σ(*I*)
graphite	*R*_int_ = 0.024
Detector resolution: 8.4353 pixels mm^-1^	θ_max_ = 25.0°, θ_min_ = 3.3°
ω scan	*h* = −28→28
Absorption correction: multi-scan (*CrysAlis RED*; Oxford Diffraction, 2009)	*k* = −7→7
*T*_min_ = 0.965, *T*_max_ = 1.000	*l* = −17→29
17425 measured reflections	

### Refinement

**Table:**

Refinement on *F*^2^	Primary atom site location: structure-invariant direct methods
Least-squares matrix: full	Secondary atom site location: difference Fourier map
*R*[*F*^2^ > 2σ(*F*^2^)] = 0.033	Hydrogen site location: inferred from neighbouring sites
*wR*(*F*^2^) = 0.080	H-atom parameters constrained
*S* = 0.96	*w* = 1/[σ^2^(*F*_o_^2^) + (0.0466*P*)^2^] where *P* = (*F*_o_^2^ + 2*F*_c_^2^)/3
3315 reflections	(Δ/σ)_max_ < 0.001
236 parameters	Δρ_max_ = 0.20 e Å^−^^3^
0 restraints	Δρ_min_ = −0.20 e Å^−^^3^

### Special details

**Table:**

Geometry. All e.s.d.'s (except the e.s.d. in the dihedral angle between two l.s. planes) are estimated using the full covariance matrix. The cell e.s.d.'s are taken into account individually in the estimation of e.s.d.'s in distances, angles and torsion angles; correlations between e.s.d.'s in cell parameters are only used when they are defined by crystal symmetry. An approximate (isotropic) treatment of cell e.s.d.'s is used for estimating e.s.d.'s involving l.s. planes.
Refinement. Refinement of *F*^2^ against ALL reflections. The weighted *R*-factor *wR* and goodness of fit *S* are based on *F*^2^, conventional *R*-factors *R* are based on *F*, with *F* set to zero for negative *F*^2^. The threshold expression of *F*^2^ > 2σ(*F*^2^) is used only for calculating *R*-factors(gt) *etc*. and is not relevant to the choice of reflections for refinement. *R*-factors based on *F*^2^ are statistically about twice as large as those based on *F*, and *R*- factors based on ALL data will be even larger.

### Fractional atomic coordinates and isotropic or equivalent isotropic displacement parameters (Å^2^)

**Table:**

	*x*	*y*	*z*	*U*_iso_*/*U*_eq_	
O1	0.01323 (3)	0.97763 (12)	0.09694 (3)	0.0262 (2)	
H1	0.0440	1.0301	0.1163	0.039*	
C1	−0.05610 (5)	0.71728 (17)	0.10148 (5)	0.0194 (3)	
C2	−0.08736 (5)	0.73762 (19)	0.03965 (5)	0.0238 (3)	
H2A	−0.0807	0.8790	0.0268	0.029*	
H2B	−0.0715	0.6344	0.0187	0.029*	
C3	−0.15200 (5)	0.70122 (19)	0.02840 (5)	0.0251 (3)	
H3	−0.1710	0.7147	−0.0120	0.030*	
C4	−0.17747 (5)	0.86215 (19)	0.05970 (5)	0.0267 (3)	
H4A	−0.1717	1.0049	0.0471	0.032*	
H4B	−0.2192	0.8381	0.0525	0.032*	
C5	−0.14785 (5)	0.84099 (17)	0.12124 (5)	0.0225 (3)	
H5	−0.1640	0.9466	0.1420	0.027*	
C6	−0.15777 (5)	0.62180 (17)	0.14074 (5)	0.0246 (3)	
H6A	−0.1994	0.5969	0.1341	0.030*	
H6B	−0.1391	0.6087	0.1808	0.030*	
C7	−0.13269 (5)	0.46028 (18)	0.10926 (5)	0.0244 (3)	
H7	−0.1395	0.3168	0.1218	0.029*	
C8	−0.06786 (5)	0.49578 (17)	0.12020 (5)	0.0232 (3)	
H8A	−0.0519	0.3902	0.0999	0.028*	
H8B	−0.0487	0.4791	0.1600	0.028*	
C9	−0.16199 (5)	0.48078 (19)	0.04750 (5)	0.0279 (3)	
H9A	−0.2036	0.4547	0.0401	0.034*	
H9B	−0.1461	0.3760	0.0269	0.034*	
C10	−0.08327 (5)	0.87808 (17)	0.13238 (5)	0.0216 (3)	
H10A	−0.0646	0.8674	0.1725	0.026*	
H10B	−0.0767	1.0210	0.1204	0.026*	
C11	0.00961 (5)	0.76258 (18)	0.11266 (5)	0.0212 (3)	
C12	0.04245 (5)	0.72698 (18)	0.17460 (5)	0.0230 (3)	
H12A	0.0137	0.7129	0.1956	0.028*	
H12B	0.0631	0.5921	0.1776	0.028*	
C13	0.08517 (5)	0.89325 (18)	0.20235 (5)	0.0221 (3)	
C14	0.14410 (5)	0.8651 (2)	0.21349 (5)	0.0314 (3)	
H14	0.1586	0.7389	0.2028	0.038*	
C15	0.18185 (5)	1.0196 (2)	0.24008 (6)	0.0395 (4)	
H15	0.2220	0.9986	0.2472	0.047*	
C16	0.16163 (6)	1.2032 (2)	0.25620 (5)	0.0365 (4)	
H16	0.1877	1.3086	0.2743	0.044*	
C17	0.10354 (5)	1.2327 (2)	0.24588 (5)	0.0326 (3)	
H17	0.0893	1.3582	0.2573	0.039*	
C18	0.06578 (5)	1.08006 (18)	0.21888 (5)	0.0270 (3)	
H18	0.0257	1.1033	0.2115	0.032*	
C19	0.03612 (5)	0.62720 (19)	0.07513 (5)	0.0269 (3)	
H19A	0.0262	0.4791	0.0792	0.032*	
H19B	0.0183	0.6678	0.0363	0.032*	
C20	0.10058 (5)	0.64448 (19)	0.08689 (5)	0.0246 (3)	
C21	0.13650 (5)	0.4812 (2)	0.11175 (5)	0.0289 (3)	
H21	0.1203	0.3564	0.1213	0.035*	
C22	0.19554 (5)	0.4986 (2)	0.12276 (6)	0.0325 (3)	
H22	0.2195	0.3857	0.1396	0.039*	
C23	0.21958 (5)	0.6795 (2)	0.10939 (5)	0.0323 (3)	
H23	0.2601	0.6922	0.1174	0.039*	
C24	0.18452 (5)	0.8419 (2)	0.08428 (5)	0.0324 (3)	
H24	0.2009	0.9664	0.0748	0.039*	
C25	0.12569 (5)	0.8235 (2)	0.07287 (5)	0.0287 (3)	
H25	0.1019	0.9355	0.0551	0.034*	

### Atomic displacement parameters (Å^2^)

**Table:**

	*U*^11^	*U*^22^	*U*^33^	*U*^12^	*U*^13^	*U*^23^
O1	0.0248 (5)	0.0256 (5)	0.0256 (5)	−0.0059 (4)	0.0027 (4)	0.0034 (4)
C1	0.0207 (6)	0.0180 (6)	0.0199 (7)	0.0004 (5)	0.0061 (5)	−0.0003 (5)
C2	0.0221 (6)	0.0273 (7)	0.0218 (8)	0.0001 (5)	0.0058 (6)	−0.0006 (6)
C3	0.0211 (6)	0.0317 (7)	0.0206 (8)	0.0003 (5)	0.0025 (5)	−0.0015 (6)
C4	0.0204 (6)	0.0260 (7)	0.0323 (8)	0.0034 (5)	0.0049 (6)	0.0029 (6)
C5	0.0221 (6)	0.0195 (6)	0.0272 (8)	0.0048 (5)	0.0092 (6)	−0.0014 (5)
C6	0.0197 (6)	0.0262 (7)	0.0293 (8)	0.0006 (5)	0.0091 (6)	0.0017 (6)
C7	0.0234 (6)	0.0168 (6)	0.0345 (8)	−0.0006 (5)	0.0105 (6)	0.0005 (6)
C8	0.0228 (6)	0.0199 (6)	0.0277 (8)	0.0022 (5)	0.0083 (6)	−0.0001 (6)
C9	0.0192 (6)	0.0286 (7)	0.0357 (9)	−0.0032 (5)	0.0072 (6)	−0.0099 (6)
C10	0.0233 (6)	0.0190 (6)	0.0225 (7)	0.0004 (5)	0.0065 (5)	0.0000 (5)
C11	0.0222 (6)	0.0209 (6)	0.0203 (7)	−0.0009 (5)	0.0057 (5)	0.0009 (5)
C12	0.0225 (6)	0.0236 (6)	0.0228 (7)	0.0016 (5)	0.0059 (5)	0.0004 (6)
C13	0.0244 (6)	0.0277 (7)	0.0138 (7)	0.0011 (5)	0.0047 (5)	0.0023 (5)
C14	0.0253 (7)	0.0422 (8)	0.0253 (8)	0.0041 (6)	0.0046 (6)	−0.0077 (7)
C15	0.0238 (7)	0.0638 (10)	0.0297 (9)	−0.0067 (7)	0.0054 (6)	−0.0108 (8)
C16	0.0416 (8)	0.0420 (8)	0.0241 (8)	−0.0154 (7)	0.0062 (7)	−0.0061 (7)
C17	0.0432 (8)	0.0280 (7)	0.0233 (8)	0.0000 (6)	0.0038 (6)	−0.0012 (6)
C18	0.0274 (7)	0.0306 (7)	0.0210 (8)	0.0036 (5)	0.0034 (6)	0.0006 (6)
C19	0.0221 (6)	0.0350 (7)	0.0240 (8)	−0.0010 (5)	0.0070 (6)	−0.0058 (6)
C20	0.0226 (6)	0.0337 (7)	0.0187 (7)	−0.0002 (5)	0.0076 (5)	−0.0076 (6)
C21	0.0299 (7)	0.0269 (7)	0.0323 (8)	0.0002 (5)	0.0126 (6)	−0.0065 (6)
C22	0.0274 (7)	0.0351 (8)	0.0346 (9)	0.0096 (6)	0.0082 (6)	−0.0060 (7)
C23	0.0193 (6)	0.0439 (8)	0.0338 (9)	0.0003 (6)	0.0077 (6)	−0.0077 (7)
C24	0.0283 (7)	0.0403 (8)	0.0313 (9)	−0.0021 (6)	0.0129 (6)	0.0021 (7)
C25	0.0256 (7)	0.0394 (8)	0.0224 (8)	0.0046 (6)	0.0086 (6)	0.0046 (6)

### Geometric parameters (Å, °)

**Table:**

O1—C11	1.4414 (13)	C11—C19	1.5500 (16)
O1—H1	0.8400	C11—C12	1.5620 (16)
C1—C2	1.5397 (16)	C12—C13	1.5144 (15)
C1—C8	1.5454 (15)	C12—H12A	0.9900
C1—C10	1.5459 (15)	C12—H12B	0.9900
C1—C11	1.5680 (15)	C13—C18	1.3906 (16)
C2—C3	1.5331 (15)	C13—C14	1.3910 (15)
C2—H2A	0.9900	C14—C15	1.3876 (17)
C2—H2B	0.9900	C14—H14	0.9500
C3—C4	1.5296 (16)	C15—C16	1.3774 (19)
C3—C9	1.5316 (17)	C15—H15	0.9500
C3—H3	1.0000	C16—C17	1.3741 (17)
C4—C5	1.5258 (17)	C16—H16	0.9500
C4—H4A	0.9900	C17—C18	1.3824 (17)
C4—H4B	0.9900	C17—H17	0.9500
C5—C6	1.5276 (15)	C18—H18	0.9500
C5—C10	1.5323 (15)	C19—C20	1.5136 (15)
C5—H5	1.0000	C19—H19A	0.9900
C6—C7	1.5299 (15)	C19—H19B	0.9900
C6—H6A	0.9900	C20—C25	1.3891 (16)
C6—H6B	0.9900	C20—C21	1.3929 (17)
C7—C9	1.5289 (17)	C21—C22	1.3866 (16)
C7—C8	1.5373 (15)	C21—H21	0.9500
C7—H7	1.0000	C22—C23	1.3798 (18)
C8—H8A	0.9900	C22—H22	0.9500
C8—H8B	0.9900	C23—C24	1.3801 (17)
C9—H9A	0.9900	C23—H23	0.9500
C9—H9B	0.9900	C24—C25	1.3812 (16)
C10—H10A	0.9900	C24—H24	0.9500
C10—H10B	0.9900	C25—H25	0.9500
			
C11—O1—H1	109.5	C5—C10—H10B	109.4
C2—C1—C8	107.87 (9)	C1—C10—H10B	109.4
C2—C1—C10	107.26 (9)	H10A—C10—H10B	108.0
C8—C1—C10	108.34 (9)	O1—C11—C19	107.30 (9)
C2—C1—C11	110.87 (9)	O1—C11—C12	111.20 (9)
C8—C1—C11	112.33 (9)	C19—C11—C12	110.36 (9)
C10—C1—C11	110.01 (9)	O1—C11—C1	105.31 (9)
C3—C2—C1	111.40 (9)	C19—C11—C1	111.21 (9)
C3—C2—H2A	109.3	C12—C11—C1	111.29 (9)
C1—C2—H2A	109.3	C13—C12—C11	116.98 (9)
C3—C2—H2B	109.3	C13—C12—H12A	108.1
C1—C2—H2B	109.3	C11—C12—H12A	108.1
H2A—C2—H2B	108.0	C13—C12—H12B	108.1
C4—C3—C9	109.56 (10)	C11—C12—H12B	108.0
C4—C3—C2	110.03 (10)	H12A—C12—H12B	107.3
C9—C3—C2	108.99 (9)	C18—C13—C14	117.77 (11)
C4—C3—H3	109.4	C18—C13—C12	119.81 (10)
C9—C3—H3	109.4	C14—C13—C12	122.40 (11)
C2—C3—H3	109.4	C15—C14—C13	120.59 (12)
C5—C4—C3	108.84 (9)	C15—C14—H14	119.7
C5—C4—H4A	109.9	C13—C14—H14	119.7
C3—C4—H4A	109.9	C16—C15—C14	120.59 (12)
C5—C4—H4B	109.9	C16—C15—H15	119.7
C3—C4—H4B	109.9	C14—C15—H15	119.7
H4A—C4—H4B	108.3	C17—C16—C15	119.53 (12)
C4—C5—C6	109.76 (10)	C17—C16—H16	120.2
C4—C5—C10	109.94 (10)	C15—C16—H16	120.2
C6—C5—C10	109.16 (9)	C16—C17—C18	120.06 (13)
C4—C5—H5	109.3	C16—C17—H17	120.0
C6—C5—H5	109.3	C18—C17—H17	120.0
C10—C5—H5	109.3	C17—C18—C13	121.46 (12)
C5—C6—C7	109.34 (9)	C17—C18—H18	119.3
C5—C6—H6A	109.8	C13—C18—H18	119.3
C7—C6—H6A	109.8	C20—C19—C11	114.97 (10)
C5—C6—H6B	109.8	C20—C19—H19A	108.5
C7—C6—H6B	109.8	C11—C19—H19A	108.5
H6A—C6—H6B	108.3	C20—C19—H19B	108.5
C9—C7—C6	109.44 (10)	C11—C19—H19B	108.5
C9—C7—C8	109.43 (10)	H19A—C19—H19B	107.5
C6—C7—C8	110.04 (10)	C25—C20—C21	117.97 (11)
C9—C7—H7	109.3	C25—C20—C19	120.90 (11)
C6—C7—H7	109.3	C21—C20—C19	121.13 (11)
C8—C7—H7	109.3	C22—C21—C20	120.84 (12)
C7—C8—C1	110.35 (9)	C22—C21—H21	119.6
C7—C8—H8A	109.6	C20—C21—H21	119.6
C1—C8—H8A	109.6	C23—C22—C21	120.17 (12)
C7—C8—H8B	109.6	C23—C22—H22	119.9
C1—C8—H8B	109.6	C21—C22—H22	119.9
H8A—C8—H8B	108.1	C22—C23—C24	119.65 (12)
C7—C9—C3	109.32 (9)	C22—C23—H23	120.2
C7—C9—H9A	109.8	C24—C23—H23	120.2
C3—C9—H9A	109.8	C23—C24—C25	120.11 (13)
C7—C9—H9B	109.8	C23—C24—H24	119.9
C3—C9—H9B	109.8	C25—C24—H24	119.9
H9A—C9—H9B	108.3	C24—C25—C20	121.25 (12)
C5—C10—C1	111.28 (9)	C24—C25—H25	119.4
C5—C10—H10A	109.4	C20—C25—H25	119.4
C1—C10—H10A	109.4		
			
C8—C1—C2—C3	58.61 (12)	C8—C1—C11—C19	68.28 (13)
C10—C1—C2—C3	−57.89 (12)	C10—C1—C11—C19	−170.96 (10)
C11—C1—C2—C3	−178.01 (9)	C2—C1—C11—C12	−175.98 (9)
C1—C2—C3—C4	60.09 (13)	C8—C1—C11—C12	−55.21 (12)
C1—C2—C3—C9	−60.06 (12)	C10—C1—C11—C12	65.55 (12)
C9—C3—C4—C5	60.47 (12)	O1—C11—C12—C13	−17.22 (14)
C2—C3—C4—C5	−59.33 (12)	C19—C11—C12—C13	101.75 (11)
C3—C4—C5—C6	−60.57 (11)	C1—C11—C12—C13	−134.29 (10)
C3—C4—C5—C10	59.52 (12)	C11—C12—C13—C18	77.21 (14)
C4—C5—C6—C7	60.41 (12)	C11—C12—C13—C14	−104.43 (13)
C10—C5—C6—C7	−60.15 (13)	C18—C13—C14—C15	−0.20 (19)
C5—C6—C7—C9	−59.80 (12)	C12—C13—C14—C15	−178.59 (12)
C5—C6—C7—C8	60.48 (13)	C13—C14—C15—C16	0.4 (2)
C9—C7—C8—C1	60.66 (12)	C14—C15—C16—C17	0.1 (2)
C6—C7—C8—C1	−59.63 (13)	C15—C16—C17—C18	−0.8 (2)
C2—C1—C8—C7	−58.44 (12)	C16—C17—C18—C13	1.0 (2)
C10—C1—C8—C7	57.36 (12)	C14—C13—C18—C17	−0.49 (18)
C11—C1—C8—C7	179.08 (10)	C12—C13—C18—C17	177.94 (12)
C6—C7—C9—C3	59.81 (12)	O1—C11—C19—C20	71.11 (13)
C8—C7—C9—C3	−60.84 (12)	C12—C11—C19—C20	−50.21 (13)
C4—C3—C9—C7	−60.35 (12)	C1—C11—C19—C20	−174.22 (10)
C2—C3—C9—C7	60.09 (12)	C11—C19—C20—C25	−72.41 (15)
C4—C5—C10—C1	−60.40 (12)	C11—C19—C20—C21	107.99 (13)
C6—C5—C10—C1	60.05 (13)	C25—C20—C21—C22	0.84 (19)
C2—C1—C10—C5	58.06 (12)	C19—C20—C21—C22	−179.55 (11)
C8—C1—C10—C5	−58.14 (12)	C20—C21—C22—C23	0.34 (19)
C11—C1—C10—C5	178.72 (9)	C21—C22—C23—C24	−0.9 (2)
C2—C1—C11—O1	63.42 (11)	C22—C23—C24—C25	0.3 (2)
C8—C1—C11—O1	−175.82 (9)	C23—C24—C25—C20	0.9 (2)
C10—C1—C11—O1	−55.05 (12)	C21—C20—C25—C24	−1.47 (19)
C2—C1—C11—C19	−52.49 (12)	C19—C20—C25—C24	178.92 (11)

### Hydrogen-bond geometry (Å, °)

**Table:**

*Cg*1 is the centroid of C20–C25 ring.

**Table:**

*D*—H···*A*	*D*—H	H···*A*	*D*···*A*	*D*—H···*A*
C14—H14···Cg1	0.95	2.70	3.3172 (13)	123
